# Development of In Vitro Parkinson's Disease Model Mediated by MPP+ and α‐Synuclein Using Wharton's Jelly Mesenchymal Stem Cells

**DOI:** 10.1111/cns.70299

**Published:** 2025-04-22

**Authors:** Naisarg Gamit, Manasi Patil, B. S. Soumya, Arun Dharmarajan, Sudha Warrier

**Affiliations:** ^1^ Division of Cancer Stem Cells and Cardiovascular Regeneration, Manipal Institute of Regenerative Medicine Manipal Academy of Higher Education (MAHE) Bangalore India; ^2^ School of Human Sciences The University of Western Australia Nedlands Western Australia Australia; ^3^ Curtin Medical School Curtin University Perth Western Australia Australia; ^4^ Department of Biotechnology, Faculty of Biomedical Sciences and Technology Sri Ramachandra Institute of Higher Education and Research Chennai India

**Keywords:** α‐Synuclein, A53T, mesenchymal stem cells, MPP+, nitrite, Parkinson's disease, ROS, WJMSCs

## Abstract

**Main Problem:**

The mechanism behind Parkinson's disease (PD) is still unclear, and a cure to stop its progression is yet to be found. This is mainly due to the lack of effective human PD models. To address this, we generated an in vitro PD model using Wharton's jelly‐derived mesenchymal stem cells (WJMSCs).

**Methods:**

WJMSCs were isolated from the umbilical cord using an enzymatic method. MSCs were characterized by RT‐PCR, immunofluorescence, and trilineage differentiation. MSCs were differentiated into dopaminergic neuron‐like cells (DAN) and further degenerated by treating them with either MPP+ iodide or the A53T mutated α‐synuclein variant. Gene expression analysis by qRT‐PCR and protein analysis by immunofluorescence, flow cytometry, and ELISA were performed. Assays to measure LDH, ROS, NO, GSH, and mitochondrial membrane potential were also performed after degeneration.

**Results:**

WJMSCs were positive for MSC markers and were able to differentiate into adipocytes, chondrocytes, and osteocytes. DAN obtained after the differentiation of WJMSCs for 48 h expressed neuronal markers such as synapsin 1, neuropilin, neurofilament, and MAPT along with dopaminergic markers such as Nurr1, DAT, TH, DDC, and KCNJ6 and were functionally active. Upon degeneration of DAN by MPP+ or A53T, elevated levels of SNCA and downregulation of TH, Nurr1, DAT, and KCNJ6 were observed. Furthermore, increased expression of α‐SYN was detected at the protein level as well. Finally, reduction in mitochondrial membrane potential and GSH levels along with an increase in intracellular ROS, nitrite production, and LDH levels confirmed that the in vitro PD‐like model exhibited the molecular characteristics of PD.

**Conclusion:**

This model is rapid, cost‐efficient, and effective for understanding the molecular mechanisms of the disease and can also be used for screening of emerging therapeutics for PD.

## Graphical abstract

A cost‐effective and rapid in vitro model of Parkinson's disease was created using Wharton's Jelly‐derived mesenchymal stem cells by employing MPP+ iodide or A53T mutated α‐synuclein.

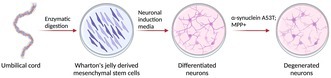

**Abbreviations**
bFGF: basic fibroblast growth factor
BSA: bovine serum albumin
cDNA: complementary DNA
DAN: dopaminergic neurons‐like cells
DAT: dopamine transporter
DDC: dopamine decarboxylase
ELISA: indirect enzyme‐linked immunosorbent assay
FBS: fetal bovine serum
GSH: reduced glutathione
IBMX: 3‐isobutyl‐1‐methylxnthin
KCNJ6: potassium inwardly rectifying channel J6
LDH: lactate dehydrogenase
MEA: multi‐electrode array
MPP+: 1‐methyl‐4‐phenylpyridinium
MPTP: 1‐methyl‐4‐phenyl‐1,2,3,6‐tetrahydropyridine
MSCs: mesenchymal stem cells
NO: nitric oxide
Nurr1: nuclear receptor related 1
PBS: phosphate buffered saline
PD: parkinson's disease
PFA: paraformaldehyde
qRT‐PCR: quantitative
RA: retinoic acid
RNA: ribonucleic acid
RNS: reactive nitrogen species
ROS: reactive oxygen species
RT‐PCR: reverse transcriptase‐polymerase chain reaction
Shh: sonic hedgehog
SNCA: α‐synuclein gene
SNpc: substantia nigra pars compacta
TH: tyrosine hydroxylase
TMB: tetramethylbenzidine
Tuj1: beta Tubulin
VMAT: vesicular monoamine transporter
WJMSCs: Wharton's jelly‐derived mesenchymal stem cells
WT: wild‐type
α‐SYN: alpha‐synuclein
βME: β‐mercaptoethanol

## 1 Introduction

Parkinson's disease (PD) stands as the 2nd most prevalent neurodegenerative disorder globally, following Alzheimer's disease. The gradual loss of dopaminergic neurons in the pars compacta of substantia nigra (SNpc) contributes to both primary (bradykinesia, resting tremor, postural instability, and rigidity) and secondary (unwanted acceleration, hypomimia, and freezing) motor symptoms [1, 2]. Depletion of dopamine in striatal projections disrupts the cerebral neuronal system involved in motor functions [3]. PD diagnosis is often based on the presence of cytoplasmic (Lewy bodies) and neuritic (Lewy neurites) alpha‐synuclein (α‐SYN) inclusions in neurons and key areas of the central nervous system [4, 5]. However, the precise mechanisms underlying this disease remain unclear, and a definitive cure to halt disease progression is still lacking. This is primarily due to the absence of robust PD models in the human context. While various cellular models and animal models are utilized to explore PD mechanisms and identify potential neuroprotective agents, they each possess limitations and are still incomplete [6, 7].

Human mesenchymal stem cells (MSCs) present significant potential for establishing in vitro models of neurodegenerative disorders including PD due to their inherent nature as stem cells. MSCs are highly regarded in regenerative medicine due to their non‐tumorigenic and non‐immunogenic properties, as well as their capacity to differentiate into multiple lineages of human organs. Dopaminergic neurons derived from stem cells are widely utilized for exploring neuronal molecular mechanisms, studying PD, investigating cell replacement therapy, and screening compounds targeting PD [8]. Previous research has shown that Wharton's jelly‐derived mesenchymal stem cells (WJMSCs) have the potential to differentiate into neuron‐like cells and express neuronal genes when exposed to combinations of compounds, small molecules, and growth factors [9, 10].

PD cases manifest as either sporadic or familial. Sporadic cases stem from factors like age and exposure to environmental toxins such as neurotoxicants like MPTP, as well as pesticides like rotenone and paraquat [11]. Familial PD includes autosomal recessive and dominant forms. Autosomal recessive PD is linked to mutations in PINK1 and Parkin, while autosomal dominant PD involves mutations in SNCA, LRRK2, and GBA [12, 13]. Rare PD forms result from SNCA mutations involving gene duplication/triplication or point mutations such as A53T, A30P, E46K, H50Q, and G51D [12, 14, 15, 16, 17]. Understanding both PD forms is essential for grasping disease mechanisms and developing treatments. Typically, Sporadic PD models use neurotoxins like 1‐methyl‐4‐phenyl‐1,2,3,6‐tetrahydropyridine (MPTP), rotenone, and 6‐OHDA, while familial PD models overexpress wild type or mutated genes such as SNCA, LRRK2, Parkin, and PINK1 [18, 19, 20, 21]. In vitro models often utilize 1‐methyl‐4‐phenylpyridinium (MPP+), which is an active metabolite of MPTP, to mimic its neurotoxic effects. On the other hand, α‐SYN aggregation into Lewy bodies is a hallmark of both familial and sporadic PD, with mutations or multiplications in the SNCA gene causing inheritable familial PD [11, 21]. The factors that lead to neurodegeneration in the case of both sporadic and familial PD are oxidative stress and dysfunction of mitochondrial membrane potential among others [22, 23]. In the present study, we have isolated human perinatal MSCs, specifically WJMSCs, and differentiated them into functional dopaminergic neuron‐like cells (DAN). To induce degeneration in WJMSCs derived DAN, we employed MPP+ iodide and α‐SYN (A53T)‐induced cytotoxicity to establish PD models.

## 2 Materials and Methods

### 2.1 Isolation, Culture, and Maintenance of WJMSCs

WJMSCs were isolated, cultured, and maintained as reported previously [24]. To remove blood contaminants, the umbilical cord was properly washed in phosphate buffered saline (PBS) and cut open lengthwise from the middle to remove blood vessels. The cord was then cut into small pieces and kept in a fresh 60 mm dish. Tissue was digested by adding equal volumes of trypsin (0.25%) and type I collagenase (0.5 mg/mL) overnight at 37°C. Digested tissue was then neutralized by culture media, strained using a 70 μm strainer, and centrifuged for 20 min at 2000 rpm. Cells were washed with PBS, resuspended in media and plated in a T25 flask containing culture media at 37°C in a 5% CO_2_ incubator. Culture media contain Knockout Dulbecco's Modified Eagle Medium/Nutrient Mixture F‐12 (KO‐DMEM/F‐12), GlutaMAX (1X), Anti‐Anti (1X), and fetal bovine serum (FBS; 10%). Cells were observed under a Nikon‐Eclipse TE2000‐S (Nikon Corporation, Tokyo, Japan). WJMSCs were cryopreserved by using cryomedia composed of culture media, FBS, and DMSO (7:2:1) and stored at −196°C for further use. All the chemicals were purchased from Gibco, MA, USA if not mentioned otherwise. Ethical clearances were obtained from the Institutional Ethical Committee (IEC), Omega Multispecialty Hospital, Bangalore, India, and the Institutional Committee for Stem Cell Research (IC‐SCR), MIRM, MAHE, Bangalore, India.

### 2.2 Trilineage Differentiation

WJMSCs were differentiated into adipocytes, osteocytes, and chondrocytes as follows: (i) Adipogenesis: Dexamethasone (1 μM), 3‐isobutyl‐1‐methylxanthine (IBMX; 0.5 mM), indomethacin (100 μM) and insulin (1 μg/mL) in DMEM (Gibco, MA, USA) + 10% FBS for 15–20 days or until the appearance of lipid droplets. Cells were fixed with paraformaldehyde (PFA; 4%) and lipid droplets were stained by Oil red ‘O’. (ii) Osteogenic differentiation: dexamethasone (0.01 μM), β‐glycerophosphate (10 mM) and ascorbic acid (50 μg/mL) in DMEM + 10% FBS for 20 days. Calcium deposits were stained with 2% Alizarin Red after fixation with paraformaldehyde (PFA; 4%). (iii) Chondrogenic differentiation: Dexamethasone (1 μM), ascorbic acid (1 μM), ITS (2%), and sodium pyruvate (1%; Gibco, MA, USA) in DMEM + 10% for 20 days. Glycosaminoglycans were stained with 1% Alcian blue after the cells were fixed. Unless otherwise mentioned all the chemicals and reagents were procured from Sigma Aldrich, MO, USA. Stained cells were observed under Nikon‐Eclipse TE2000‐S (Nikon Corporation, Tokyo, Japan) or Accu‐Scope EXI‐310‐PH (Accu‐Scope, NY, USA).

### 2.3 Dopaminergic Differentiation and Degeneration

WJMSCs were plated on appropriate culture plates. After the cells attained the morphology, they were induced with neuronal induction media as per previous reports with modifications [25, 26]. Cells were incubated in DMEM supplemented with FBS (2%), containing N2 (Gibco, MA, USA), B27 (Gibco, MA, USA), basic fibroblast growth factor (bFGF; Sino Biologicals), epidermal growth factor (EGF; Biovision), retinoic acid (RA; Sigma Aldrich), and ß‐mercaptoethanol (ß‐ME; Sigma Aldrich) at 37°C in a 5% CO_2_ incubator to generate DAN. Cells were then degenerated using MPP+ iodide (MPP+; Cat# D048; Sigma Aldrich, MO, USA) or oligomerized alpha‐synuclein, A53T (A53T; Cat# S1071; Sigma Aldrich, MO, USA) by incubating the cells for 48 h. Staurosporine (Cat# 191400; MP Biomedicals, Irvine, USA) was used as a positive neurotoxic control.

### 2.4 RT‐PCR and qRT‐PCR

Total ribonucleic acid (RNA) was extracted using RNAiso Plus reagent, and the PrimeScript 1st strand cDNA synthesis kit was used to convert RNA into complementary DNA (cDNA). We then used EmeraldAmp GT PCR Master Mix to perform reverse transcriptase‐polymerase chain reaction (RT‐PCR) and resolved it on a 2% agarose gel. The gel image was captured using Imaglab software in a ChemiDoc XRS+ gel documentation system (Bio‐Rad, CA, USA). To perform quantitative RT‐PCR (qRT‐PCR) we used TB Green Premix Ex Taq II (Tli RNase H Plus) and ran the PCR in a QuantStudio 5 Real‐Time PCR Systems (Applied Biosystems, Thermo Fisher Scientific, USA). Reagents and primers were purchased from TaKaRa, Shiga, Japan, and Sigma Aldrich, Bangalore, India (Table 1), respectively. 2^−∆∆CT^ was utilized to calculate relative gene expression [27, 28].

**TABLE 1:** List of primers.

Gene	Sequence	Product length (bp)
GAPDH	F: 5′‐CGACCACTTGTCAAGCTCA‐3′	202
	R: 5′‐AGGGGAGATTCAGTGTGGT‐3′	
CD44	F: 5′‐CATCTACCCCAGCAACCCTA‐3′	271
	R: 5′‐GGTTGTGTTTGCTCCACCTT‐3′	
CD90	F: 5′‐CAAGGTCAAGTGAGCTGGGA‐3′	145
	R: 5′‐TTCACACCGAAGGCAATGGT‐3′	
CD73	F: 5′‐GCCGCTTTAGAGAATGCAAC‐3′	234
	R: 5′‐CTCGACACTTGGTGCAAAGA‐3′	
CD105	F: 5′‐CCACTAGCCAGGTCTCGAAG‐3′	192
	R: 5′‐GATGCAGGAAGACACTGCTG‐3′	
CD34	F: 5′‐CTACAACACCTAGTACCCTTGGA‐3′	185
	R: 5′‐GGTGAACACTGTGCTGATTACA‐3′	
CD45	F: 5′‐AGGTAGTAGATGTTTTCCAAGTAGTGA‐3′	127
	R: 5′‐ACTTGTCCATTCTGGGCAGGGTAG‐3′	
Nurr1	F: 5′‐GCTGCCCTGGCTATGGTCA‐3′	200
	R: 5′‐ATGCGCTGTAGCCCCTGTG‐3′	
DAT	F: 5′‐CAACCTGTACTGGCGGCTAT‐3′	234
	R: 5′‐GCATAGGCCAGTTTCTCTCG‐3′	
TH	F: 5′‐GCGCAGGAAGCTGATTGCTG‐3′	200
	R: 5′‐TGTCTTCCCGGTAGCCGCTG‐3′	
Synapsin I	F: 5′‐TCAGACCTTCTACCCCAATCA‐3′	127
	R: 5′‐GTCCTGGAAGTCATGCTGGT‐3′	
Neuropilin	F: 5′‐GAAGGCAACAACAACTATGA‐3′	354
	R: 5′‐ATGCTCCCAGTGGCAGAATG‐3′	
Neurofilament	F: 5′‐CGCTATGCAGGACACGATCA‐3′	255
	R: 5′‐CTGGTCTGTAAACCGCCGTA‐3′	
DDC	F: 5′‐GAACAGACTTAACGGGAGCCTTT‐3′	218
	R: 5′‐ATTGCCGGTAGTCAGTGATAAGC‐3′	
KCNJ6	F: 5′‐GCTACCGGGTCATCACAGAT‐3′	163
	R: 5′‐ACTGCATGGGTGGAAAAGAC‐3′	
SNCA (transcript variant 1–3, 5–8)	F: 5′‐CCAGTTGGGCAAGAATGAAGAA‐3′	110
	R: 5′‐CTTGATACCCTTCCTCAGAAGGC‐3′	

### 2.5 Immunocytochemistry Staining

Cells were fixed and permeabilized using PFA (4%) and Triton X‐100 for 15 min each, followed by blocking using bovine serum albumin (BSA; 3%) at room temperature (RT) for 30 mins. For surface markers, permeabilization was not done. Cells were further incubated in conjugated/unconjugated primary antibodies, either overnight at 4°C or for 4 h at RT. Cells were then washed and incubated in secondary antibody for 2 h at RT, followed by DAPI (1 μg/mL; Life Technologies, CA, USA) to counterstain the nucleus for 10 min at RT (Table S1). After each step, cells were washed with PBST. Stained cells were visualized under Olympus IX73 (Olympus, Tokyo, Japan) fluorescent microscope or Leica THUNDER DMi8 (Leica Microsystems, Wetzlar, Germany) and processed using ImageJ/Fiji (NIH, Bethesda, MD, USA) [29].

### 2.6 Flow Cytometry

For surface markers, cells were fixed by incubating them with PFA (4%) for 15 min, followed by blocking with BSA (3%) for 30 min at RT. In the case of intracellular markers, permeabilization was carried out using Triton X‐100 (0.1%) for 15 min at RT. Cells were incubated with primary antibodies overnight at 4°C. Cells were then stained with respective secondary antibodies for 1 h (Table S1). Cells were washed thrice after every step with PBST (1X). Isotypes were kept the same for CD73 and CD44 and for CD34 and CD45. Flow cytometry analysis was carried out using BD FACSCalibur, BD FACSLyric, or BD Accuri C6 flow cytometer (BD Biosciences, CA, USA) [29, 30].

### 2.7 Enzyme‐Linked Immunosorbent Assay

Cells were lysed using RIPA buffer containing 1 mM phenylmethanesulfonyl fluoride (PMSF) and 1X protease inhibitor cocktail. This suspension was stored at −80°C overnight, followed by centrifugation at top speed for 15 min at 4°C. Cell lysate was collected and frozen till further use. The concentration of protein was quantified by the Bradford method and measured at 450 nm.

#### 2.7.1 Indirect ELISA

A 10 μg/mL concentration was used to perform indirect ELISA. The protein sample was incubated in a 96‐well plate precoated with coating buffer overnight at 4°C. Wells were then blocked with BSA and incubated at RT for 2 h. The primary antibody was added and further incubated overnight at 4°C on a rocker. Thereafter, the secondary antibody was added, followed by a 2 h incubation at RT (Table S1). Wells were washed thrice with TBST (1X) after each step. Lastly, 3,3′,5,5′‐tetramethylbenzidine (TMB) substrate (Sigma Aldrich, MO, USA) was added to each well and incubated for 30 min at RT in the dark. 1 M sulfuric acid was used to stop the reaction, and absorbance was recorded in a SpectraMax M5 multi‐mode microplate reader (Molecular Devices, CA, USA) at 450 nm, which was equipped with SoftMax Pro 7 data acquisition and analysis software [31].

#### 2.7.2 Dopamine ELISA

Competitive ELISA for dopamine estimation was performed using the Dopamine ELISA Kit (Cat# E‐EL‐0046, Elabscience Biotechnology Inc., USA) according to the manufacturer's protocol. Absorbance was recorded in an EnSpire multimode plate reader (PerkinElmer, MA, USA) at 450 nm.

### 2.8 Multielectrode Array

WJMSCs were plated on a 0.1% gelatin‐coated 60‐electrode multi‐electrode array (MEAs; 60MEA200/10iR‐TI‐gr). Upon the attachment of cells, the MEA with undifferentiated WJMSCs was placed on the MEA workstation, that is, MEA2100‐Lite‐System, for recording electric potential changes and extracellular field potential using Multi Channel Experimenter (v2.15.0). The MEA workstation comprised the MEA2100 headstage (MEA2100HS‐60) connected with the MCS‐Lite interface board 3.0 (MCS‐Lite‐IFB). WJMSCs were then induced with neuronal induction media for 48 h, and the electric potential was recorded again. Data was analyzed using Multi Channel Analyzer (v15.0). Filtered data from three experimental replicates (i.e., from three electrodes) were used to represent the spikes in a pictorial form. MEA, the MEA workstation, and the software used were from Multi Channel Systems, Reutlingen, Germany.

### 2.9 Neurite Outgrowth Staining

Neurite outgrowth staining kit (Molecular Probes, Life Technologies, CA, USA) was used to visualize the viability and membrane of cells. Media was removed from the cells and washed with PBS. Cell viability indicator and cell membrane stain were added in buffer containing 4% formaldehyde and added in the wells and incubated at 37°C for 20 min followed by the addition of 1X background suppression dye and visualization of the cells under a fluorescent microscope Olympus IX73 (Olympus, Tokyo, Japan). Neurites were measured in ImageJ/Fiji with the NeuronJ plugin.

### 2.10 MTT Cell Viability Assay

WJMSCs were plated in a 96‐well plate (8000 cells/well) and once they gained morphology, they were differentiated and degenerated with MPP, A53T, and staurosporine. MTT reagent (5 mg/mL; Sigma Aldrich, MO, USA) was added, and the cells were incubated for 3 h at 37°C until the formation of formazan crystals. Crystals were then dissolved by adding DMSO and further incubating for 10 min at room temperature. The absorbance was measured at 495 nm [32].

### 2.11 Determination of Apoptosis by Caspase‐3/7 Detection Assay

To determine apoptosis after treatment of DAN with MPP+, A53T, and staurosporine, caspase‐3/7 activity was estimated using the CellEvent caspase‐3/7 green detection reagent (Thermo fisher, USA) as per the manufacturer's instructions. Cells were plated in a 96‐well plate at a density of 8000 cells/well. The fluorescence intensity was measured at Ex/Em = 502/530 nm [33].

### 2.12 Measurement of LDH

Lactate dehydrogenase (LDH) release was estimated using the PicoProbe LDH‐Cytotoxicity fluorometric assay kit (BioVision, Milpitas, CA, USA) as per the manufacturer's instructions. WJMSCs were plated in a 96‐well plate at a density of 8000 cells/well. For the experiment, 50 μL of LDH reaction mix was prepared, containing 2 μL LDH substrate mix, 2.5 μL PicoProbe, and 45.5 μL LDH assay buffer. To 50 μL of the supernatant from the differentiated and degenerated conditions, 50 μL of reaction mix was added. After gently shaking the plate for 10 min at RT, fluorescence was measured at Ex/Em = 535/587 nm using a multimode microplate reader [29].

### 2.13 Determination of Mitochondrial Membrane Potential

To assess the mitochondrial membrane potential after degeneration, WJMSCs (8000 cells/well) were plated in a 96‐well plate and further differentiated and degenerated. Supernatant was discarded from the wells, and the cells were incubated with TMRE (0.3 μM; Molecular Probes, Life Technologies, CA, USA) containing media for 30 min at 37°C in the dark. TMRE fluorescence was measured at Ex/Em = 549/574 nm [31].

### 2.14 Measurement of ROS

Generation of reactive oxygen species (ROS) was measured by employing the ROS Detection Assay Kit (BioVision, Milpitas, CA, USA). WJMSCs (8000 cells/well) were plated in a 96‐well plate and incubated for 24 h. After differentiation and degeneration of WJMSCs, the supernatant was discarded from the wells, and ROS label (1:1000 in ROS assay buffer) was added. Cells were incubated in the dark for 45 min at 37°C. Intracellular ROS release was measured by capturing the fluorescence intensity at Ex/Em = 495/529 nm [29].

### 2.15 Estimation of NO and Nitrites

Griess reagent (modified; Sigma Aldrich) was used to measure the levels of nitric oxide (NO) release after degeneration. Cells were plated in a 96‐well plate at the density of 8000 cells/well, followed by differentiation and degeneration. For the standard curve of sodium nitrite, concentrations in the range of 3.125–2000 μM were prepared. Griess reagent was added to the standards or samples in equal volumes, followed by a 15 min incubation at 37°C. Absorbance was recorded at 540 nm, and using a standard curve, the concentration of nitrite was estimated.

### 2.16 Determination of GSH Levels

Reduced glutathione (GSH) levels were estimated using a glutathione detection reagent, ThiolTracker Violet (Invitrogen, CA, USA) following the manufacturer's protocol. WJMSCs were plated in a 96‐well plate at a density of 8000 cells/well. After differentiation and degeneration, the assay was performed. A working solution of the dye at a 20 μM concentration was prepared in PBS, and 100 μL of the solution was added to the wells after discarding the spent media. Incubation was carried out at 37°C in the dark for 30 min, followed by which fluorescence was measured using a multimode microplate reader at Ex/Em = 404/526 nm.

### 2.17 Statistical Analysis

Data are represented as mean ± SD. Statistical analysis was performed using GraphPad Prism 9 (San Diego, CA, USA). The normality of the distribution was tested using the Shapiro–Wilk or D'Agostino‐Pearson omnibus (K2) test. For normally distributed data, unpaired Student's *t*‐test was used to compare two groups, whereas one‐way ANOVA was used for comparing data among multiple groups, followed by Dunnett's post hoc test. Data was considered statistically significant when *p* < 0.05.

## 3 Results

### 3.1 Isolation and Characterization of WJMSCs

**FIGURE 1:**
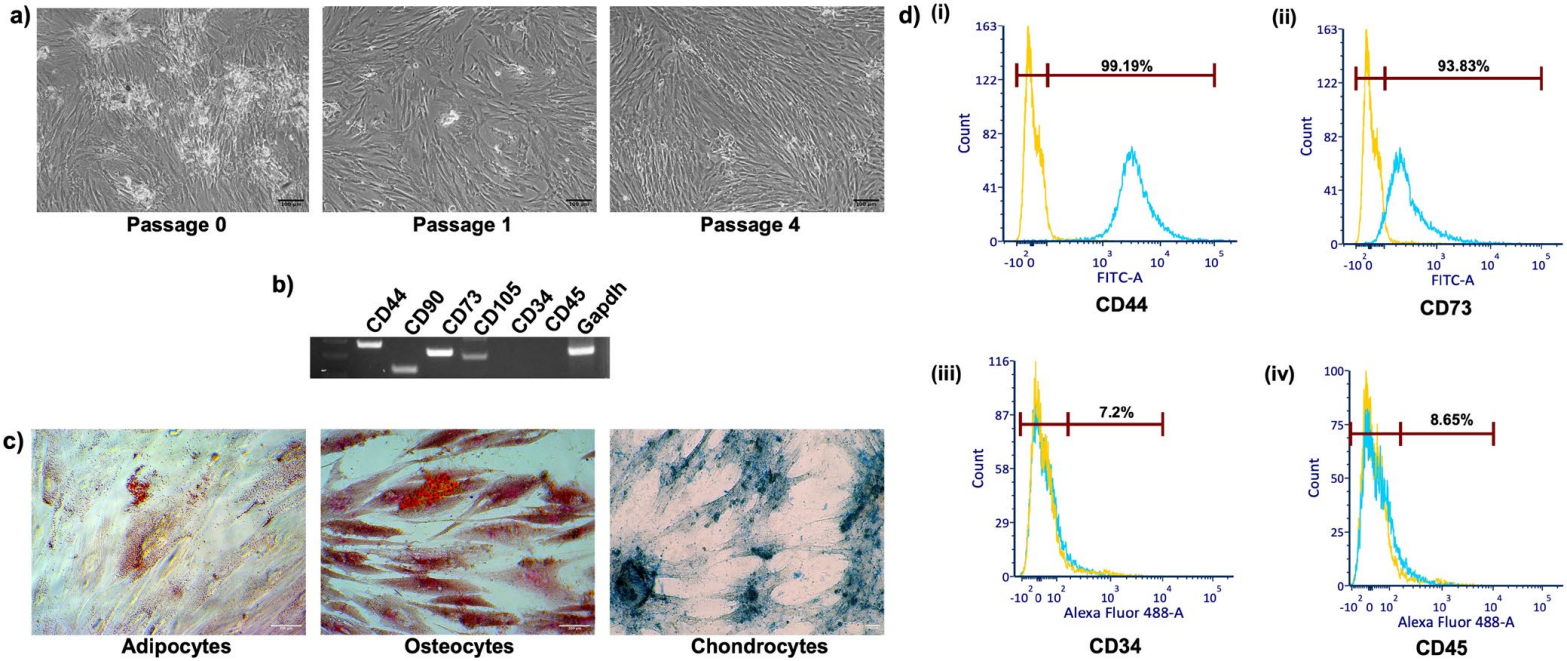
Isolation and characterization of WJMSCs. (a) Photomicrographic images of WJMSCs at passages 0, 1, 4 (Scale bar = 100 μm). (b) Characterization of WJMSCs by RT‐PCR for positive markers, CD44, CD105, CD73, CD90 and negative markers, CD34 and CD45. (c) Staining of WJMSCs after trilineage differentiation into adipocytes, osteocytes and chondrocytes by Oil Red O, Alizarin Red and Alcian Blue, respectively (Scale bar = 100 μm). (d) Flow cytometry analysis of WJMSCs for positive (i) CD44 (99.19%), (ii) CD73 (93.83%) and, negative (iii) CD34 (7.2%), (iv) CD45 (8.65%) markers.

Isolated WJMSCs displayed a fibroblast‐like spindle‐shaped morphology upon adherence to the culture dish. They reached confluence within 3–4 days and were subsequently subcultured and frozen at different passages (Figure 1a). To confirm the presence of MSCs, gene expression analysis via RT‐PCR was performed. RT‐PCR revealed positive expression of MSC markers, including CD44, CD90, CD73, and CD105, while negative expression was observed for CD34 and CD45 (Figure 1b). Given the MSCs' potential to differentiate into various lineages, we induced differentiation into adipocytes, osteocytes, and chondrocytes. WJMSCs were able to differentiate into these lineages as observed after respective staining as per the International Society of Cellular Therapy (ISCT) criteria. Oil Red O staining revealed accumulation of oil droplets in adipogenic cells, Alizarin Red staining demonstrated brown‐colored calcium deposition in osteogenic cells, and Alcian Blue staining indicated the presence of glycosaminoglycans in chondrogenic cells (Figure 1c). Cells were also confirmed for the presence of positive MSC markers and absence of negative MSC markers by flow cytometry. WJMSCs were 99.19% and 93.83% positive for CD44 and CD73, respectively (Figure 1d,i,ii) while they were negative for CD34 and CD45 (Figure 1d,iii,iv).

**FIGURE 2:**
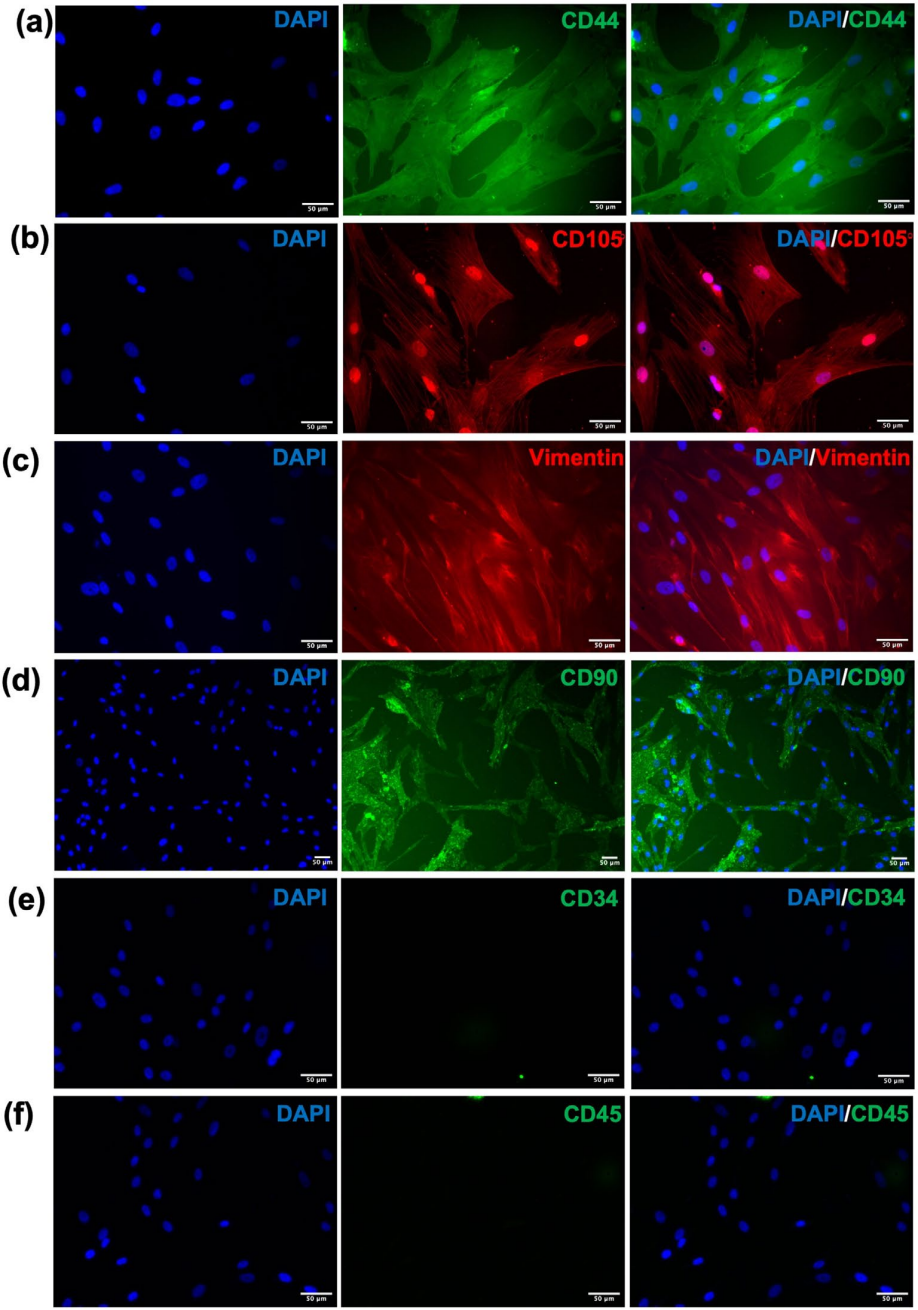
Characterization of WJMSCs by immunocytochemistry. Immunofluorescence staining for (a) CD44, (b) CD105, (c) Vimentin, (d) CD90, (e) CD34, (f) CD45 (Scale bar = 50 μm).

To further characterize these cells, immunocytochemistry was performed (Figure 2). Similar to that of gene expression, here also we observed that WJMSCs were expressing CD44, CD105, Vimentin, and CD90 (Figure 2a–d) whereas they were lacking CD34 and CD45 (Figure 2e,f). These findings collectively confirm that the isolated cells were MSCs in nature.

### 3.2 Differentiated WJMSCs Expressed Dopaminergic Markers

**FIGURE 3:**
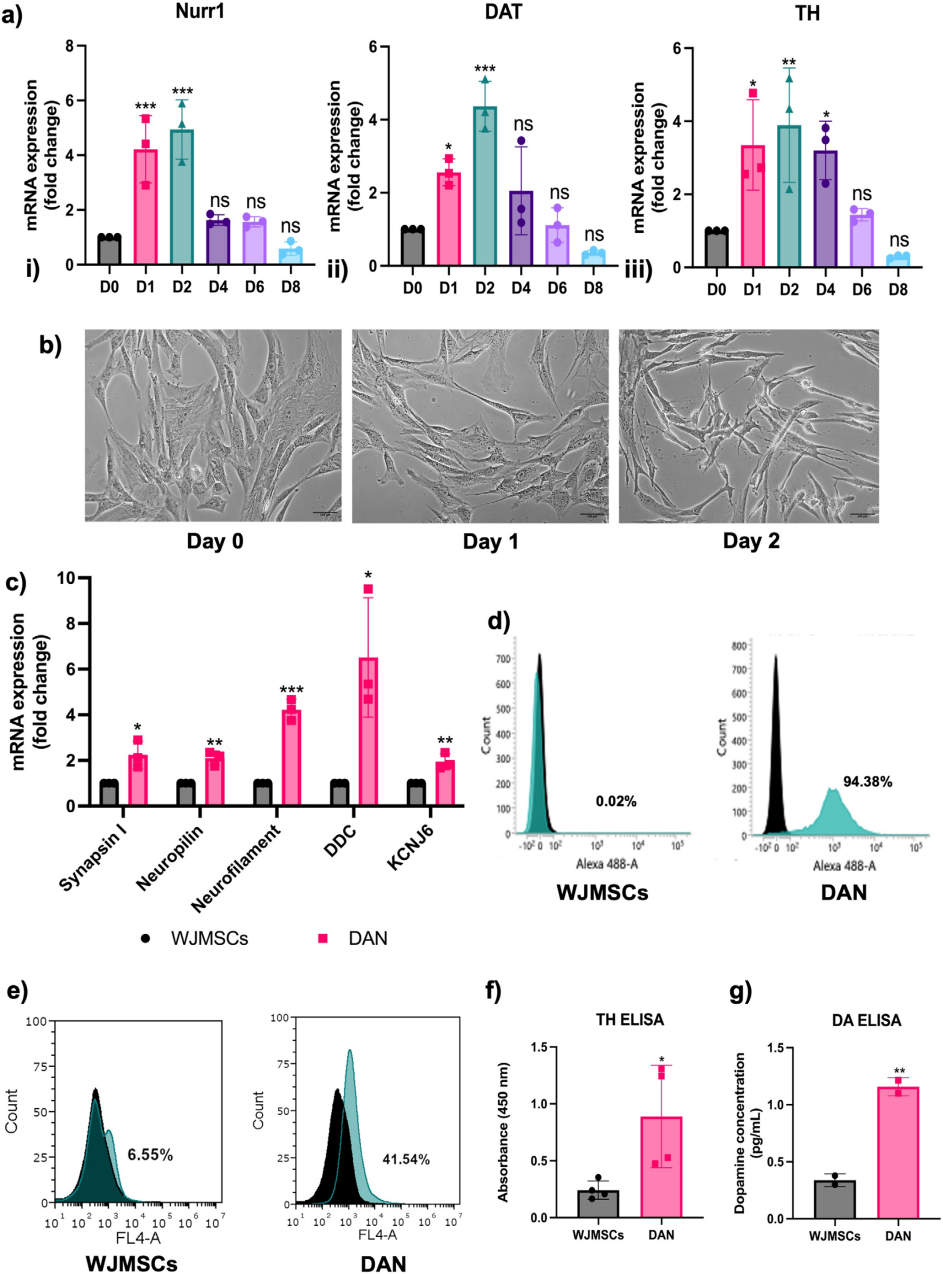
Differentiation of WJMSCs into dopaminergic neurons. (a) Gene expression analysis qRT‐PCR of dopaminergic neurons‐like cells (DAN) differentiated from WJMSCs at various intervals for Nurr1, DAT, and TH, *n* = 3, one‐way ANOVA analysis. (b) Photomicrographic images of DAN at day 1 and 2 (Scale bar = 100 μm). (c) Presence of neuronal (Synapsin I, Neuropilin, Neurofilament) and dopaminergic markers (DDC, KCNJ6) upon qRT‐PCR, *n* = 3, Student's *t*‐test analysis. Flow cytometry analysis shows DAN were positive for (d) Synapsin I (94.38%), and (e) TH (41.54%). ELISA showing (f) increased expression of TH (*n* = 4) and (g) release of dopamine in DAN as compared to WJMSCs (*n* = 2), Student's *t*‐test analysis. Data are shown as mean ± SD, *p < 0.05, **p < 0.01, ***p < 0.001.

WJMSCs were then differentiated towards the dopaminergic lineage by inducing them with neuro induction media. Cells were cultured in the neuro induction media for 8 days with media being changed every 3 days. Analysis by qRT‐PCR indicated upregulation of dopaminergic markers, including nuclear receptor related 1 (Nurr1), dopamine transporter (DAT), and tyrosine hydroxylase (TH), at 24 and 48 h post‐differentiation, gradually declining by the 8th day compared to undifferentiated WJMSCs. However, dopaminergic marker expression persisted in the differentiated cells until the 6th day (Figure 3a). For subsequent experiments, the 48 h differentiation period was chosen, and the resulting cells were designated as dopaminergic neuron‐like cells (DAN) (Figure 3b). Further gene expression analysis via qRT‐PCR demonstrated the expression of Synapsin I, Neuropilin, and Neurofilament, which are mature neuronal markers in DAN. Additionally, dopaminergic markers including dopamine decarboxylase (DDC) and potassium inwardly rectifying channel J6 (KCNJ6), encoding GIRK2, were detected (Figure 3c). At the protein level, flow cytometry, ELISA, and immunocytochemistry were performed to confirm dopaminergic differentiation. Flow cytometry revealed that 94.38% of the cells expressed Synapsin I (Figure 3d) whereas 41.54% of the cells were positive for TH (Figure 3e). ELISA analysis for TH and dopamine indicated an increase in both TH (Figure 3f) and dopamine (Figure 3g) levels upon differentiation of WJMSCs.

**FIGURE 4:**
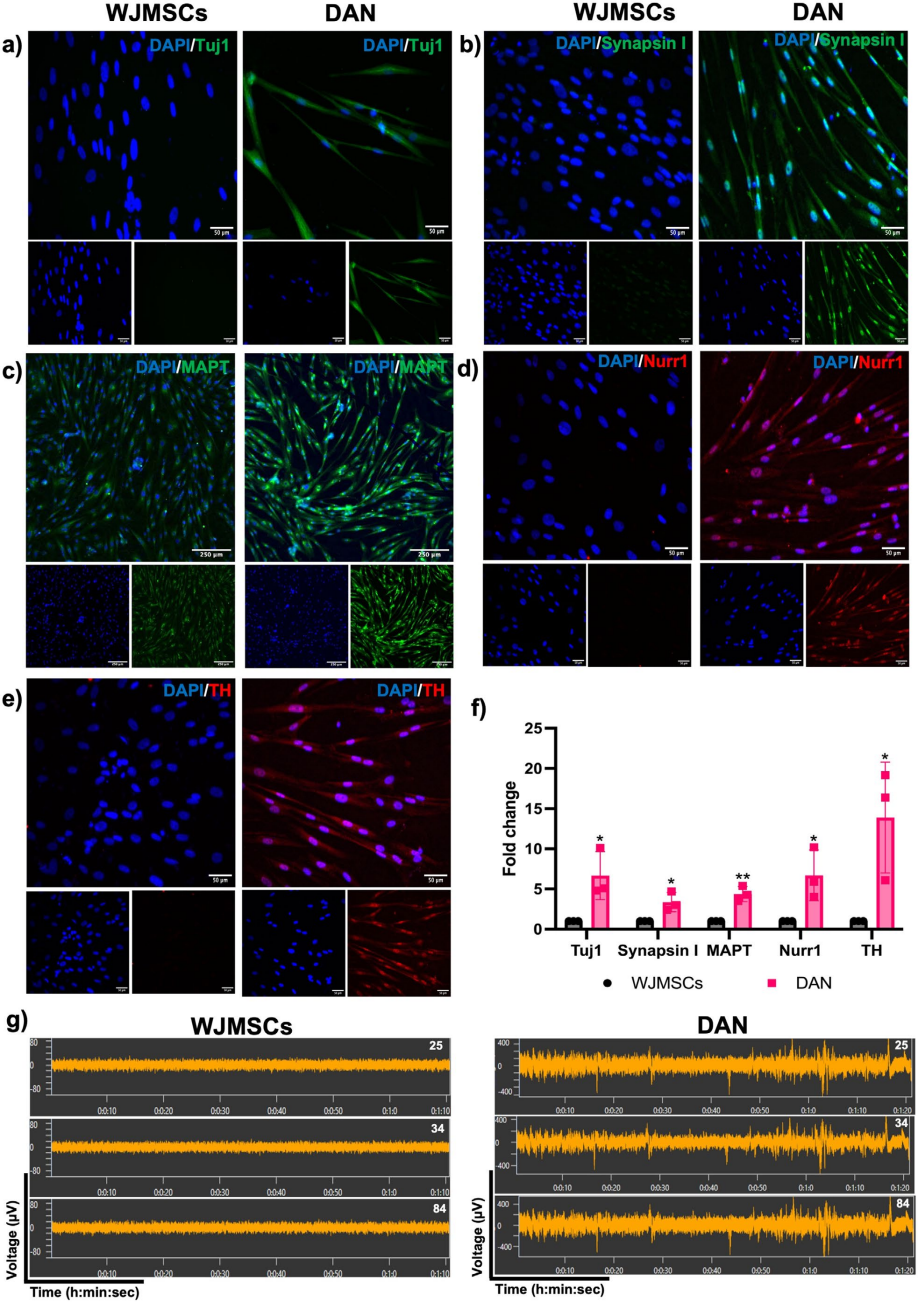
Characterization of dopaminergic neurons. Immunofluorescence staining of dopaminergic neurons showing the presence of (a) Tuj1, (b) Synapsin I, (c) MAPT (Scale bar = 250 μm), (d) Nurr1, and (e) TH (Scale bar = 50 μm). (f) Quantification of immunofluorescence images, *n* = 3, Student's *t*‐test analysis. (g) Photographic representation of spikes after differentiation of WJMSCs by multielectrode array at electrode no. 25, 34 and 84 (*n* = 3). Data are shown as mean ± SD, *p < 0.05, **p < 0.01.

Immunofluorescence staining demonstrated the expression of both neuronal markers beta Tubulin (Tuj1), Synapsin I, microtubule‐associated tau protein (MAPT), an axonal marker, and dopaminergic‐specific markers, Nurr1, TH in DAN (Figure 4a–f). Neuronal activity was recorded by MEA, which revealed that the DAN were functionally active (Figure 4g). These results confirm the successful differentiation of WJMSCs into functionally active dopaminergic‐like cells, highlighting their potential for use in modeling of neurodegenerative disease.

### 3.3 MPP And A53T Mediated Neurodegeneration of DAN

**FIGURE 5:**
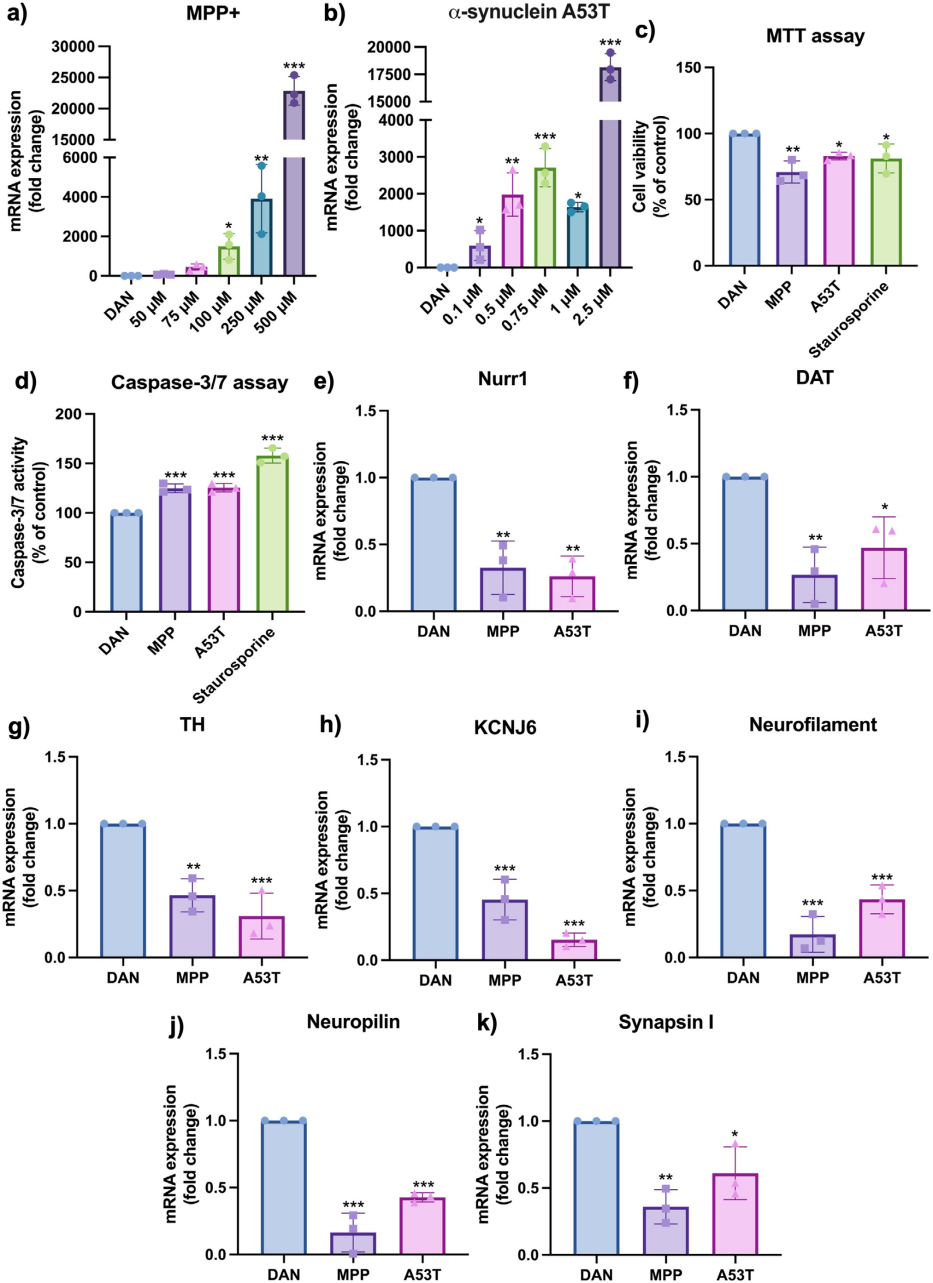
Neurodegeneration of DAN by MPP+ and α‐synuclein A53T. qRT‐PCR for SNCA after degeneration of DAN using various concentrations of (a) MPP+ and (b) α‐synuclein A53T. (c) Reduction in cell viability and (d) increased caspase‐3/7 activity upon addition of MPP+ (100 μM), A53T (0.1 μM) and staurosporine (100 nM). Reduced gene expression levels of dopaminergic markers, (e) Nurr1, (f) TH, (g) DAT, (h) KCNJ6 and neuronal markers (i), Neurofilament, (j) Neuropilin, (k) Synapsin I. *n* = 3, one‐way ANOVA analysis. Data are shown as mean ± SD, *p < 0.05, **p < 0.01, ***p < 0.001.

After confirming differentiation, neurodegeneration was induced by treating the cells with either MPP+ or A53T. MPTP crosses the blood–brain barrier and is oxidized by type B monoamine oxidase into its toxic form, MPP+, leading to dopaminergic neuron degeneration [34]. Whereas in the A53T mutation of α‐SYN, substituting alanine with threonine at the 53rd amino acid accelerates protofibril formation faster than the wild type, causing severe aggregation and neurodegeneration [12, 35, 36]. During degeneration, lower concentrations of compounds showed minimal cytotoxic effects while higher concentrations were toxic. To determine the appropriate concentration for the study, we analyzed endogenous SNCA expression, as signs of degeneration can be seen at the gene level first. Higher concentrations induced cell death and were therefore excluded. Initially, cells were incubated with varying concentrations of MPP (50, 75, 100, 250, 500 μM) and A53T (0.1, 0.5, 0.75, 1, 2.5 μM) for 48 h. A dose‐dependent increase in the expression of SNCA was observed across all concentrations (Figure 5a,b), so for subsequent experiments, 0.1 μM of A53T and 100 μM of MPP+ were selected to induce neuronal degeneration along with staurosporine (100 nM) as a positive control. After treatment, MTT cell viability and caspase‐3/7 assays were performed to assess apoptotic effects of the compounds. MTT assay showed that around 70% and 80% of cells were viable after induction with MPP+ and A53T, respectively, compared to the positive neurotoxin control, staurosporine (81%) (Figure 5c). Caspase‐3/7 activity increased by around 25% in both MPP+ and A53T conditions, while staurosporine‐treated cells exhibited a 48% increase in activity compared to DAN (Figure 5d). Following treatment with MPP+ or A53T, we also observed significant downregulation of dopaminergic‐specific markers, Nurr1, DAT, TH, and KCNJ6, as well as neuronal markers, Neurofilament, Neuropilin, and Synapsin I was observed as expected (Figure 5e–k).

**FIGURE 6:**
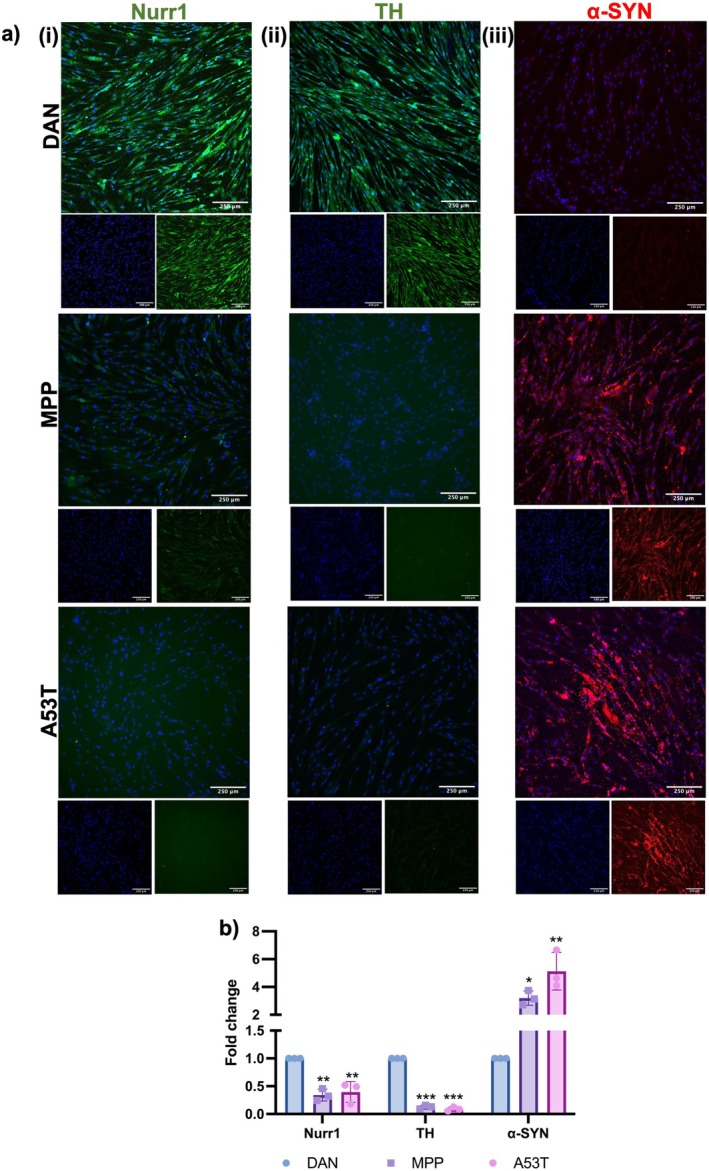
Characterization of degenerated neurons by immunofluorescence. (a) Immunofluorescence staining shows reduction in the expression of (i) Nurr1, (ii) TH and presence of (iii) α‐SYN aggregates in both MPP+ and A53T induced cells (Scale bar = 250 μm). (b) Quantification of immunofluorescence images, *n* = 3, one‐way ANOVA analysis. Data are shown as mean ± SD, *p < 0.05, **p < 0.01, ***p < 0.001.

Neurodegeneration was further confirmed by immunocytochemistry, showing results consistent with gene expression. Increased expression of α‐SYN and reduced expression of Nurr1 and TH were observed at the protein level (Figure 6).

**FIGURE 7:**
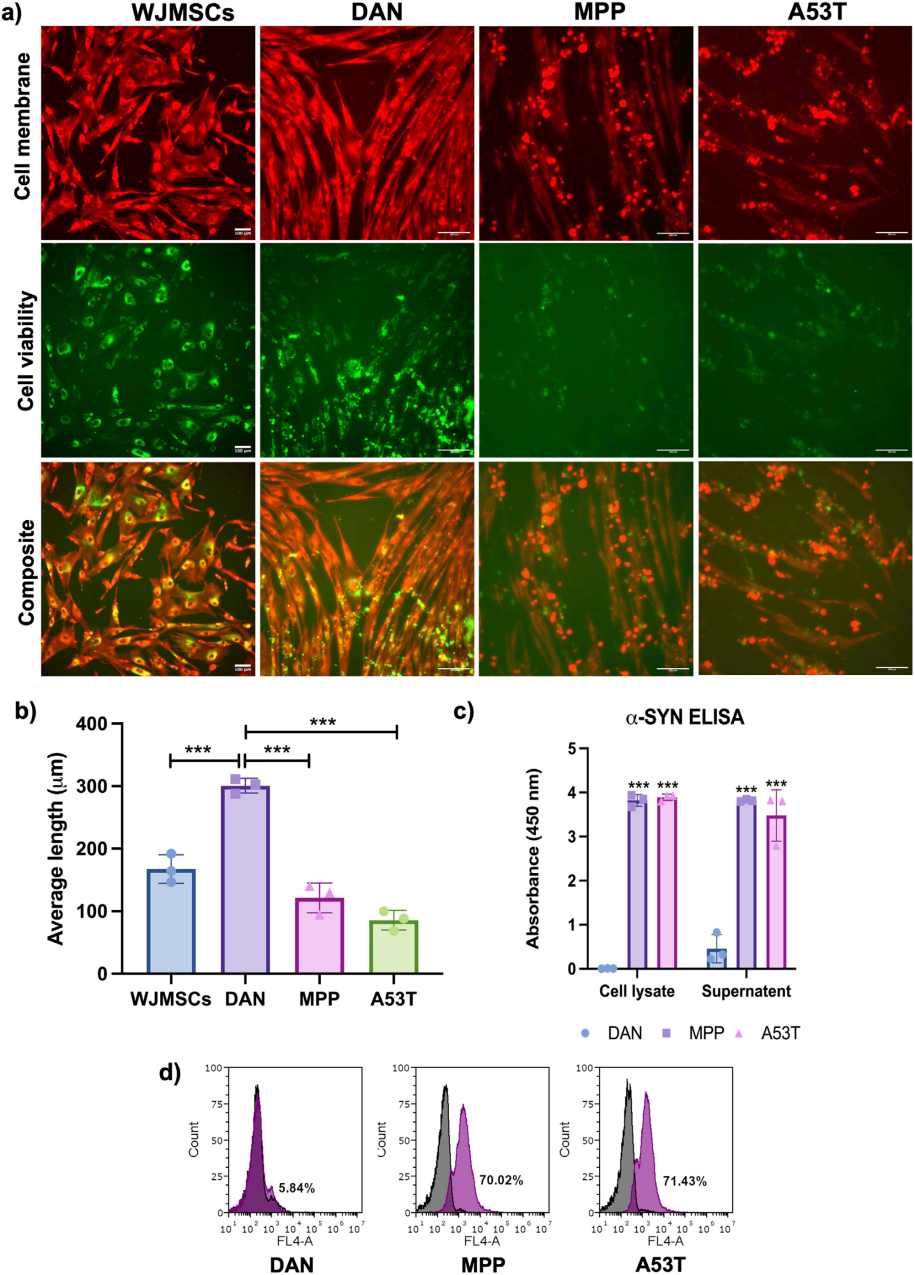
Neurite outgrowth and protein expression analysis of MPP+ and A53T induced degeneration of DAN. (a, b) DAN stained with neurite outgrowth kit for cell membrane and cell viability showed reduction of neurites upon degeneration (Scale bar = 100 μm), *n* = 3, one‐way ANOVA analysis. (c) Increased expression of α‐SYN was detected by indirect ELISA upon degeneration, *n* = 3, one‐way ANOVA analysis. (d) Flow cytometry analysis showing degenerated cells were positive α‐SYN in MPP (70.02%) and A53T (71.43%) conditions. Data are shown as mean ± SD, ***p < 0.001.

Neurites which had appeared upon differentiation of WJMSCs got reduced upon degeneration. Reduction of cell viability was also observed (Figure 7a,b). Indirect ELISA confirmed the degeneration of DAN by the presence of α‐SYN in both MPP and A53T treated conditions compared to untreated DAN (Figure 7c). Furthermore, flow cytometry revealed that 70.02% and 71.43% of cells were positive for α‐SYN upon degeneration with MPP and A53T, respectively (Figure 7d). Taken together, these findings confirm the successful induction of neurodegeneration in DAN, demonstrating their utility as an in vitro model for studying dopaminergic neuron loss.

### 3.4 MPP And A53T Increased Oxidative Stress and Destabilization of the Mitochondrial Membrane Potential

**FIGURE 8:**
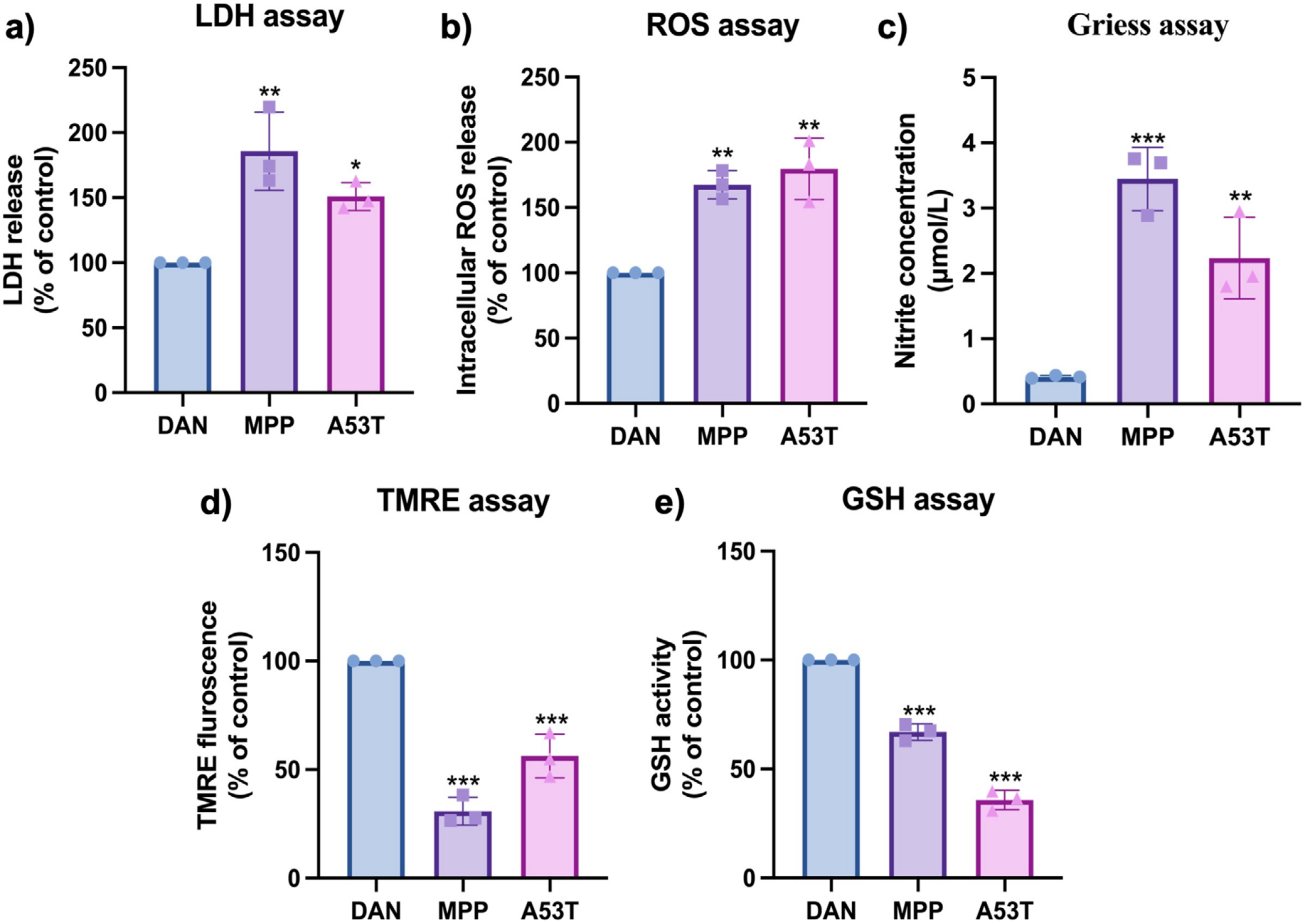
Oxidative stress analysis of MPP+ and A53T induced neurodegeneration. Increase in the levels of (a) LDH, (b) intracellular ROS release, and (c) nitrite concentration along with reduced (d) mitochondrial membrane potential estimated by TMRE and (e) GSH upon neurodegeneration. *n* = 3, one‐way ANOVA analysis. Data are shown as mean ± SD, *p < 0.05, **p < 0.01, ***p < 0.001.

Following neurodegeneration, there was an increase in LDH release compared to DAN, indicating that MPP+ and A53T induced cytotoxicity (Figure 8a). This cytotoxicity may be attributed to oxidative stress. The accumulation of oxidative damage over time contributes to PD progression, increasing neuronal vulnerability. To verify this, we conducted a ROS assay and observed a significant elevation in intracellular ROS levels compared to DAN (Figure 8b). Nitrosative stress is caused by the overproduction of NO or nitrogen dioxide. NO, being inherently unstable, tends to convert into more stable forms such as nitrites and nitrates. Alterations in NO production have been linked to cellular oxidative damage observed in PD, suggesting that dysregulation of nitric oxide synthases might contribute to the progression of the disease [37]. For this reason, we assessed NO levels using Griess assay and extrapolated the values on a sodium nitrite standard curve, which revealed higher nitrite production in both MPP+ and A53T conditions (Figure 8c).

ROS and RNS have a significant impact on mitochondria as they lack repair mechanisms, unlike the nucleus and cytoplasm, leading to mitochondrial damage by complex I inhibition [38, 39]. This was evident in both conditions, as a decrease in mitochondrial membrane potential was observed following neurodegeneration (Figure 8d). Furthermore, due to oxidative stress, the levels of GSH, which acts as an anti‐oxidant, were also reduced in neurodegenerative conditions (Figure 8e). Overall, these results highlight the role of oxidative and nitrosative stress in neurodegeneration, reinforcing their contribution to mitochondrial dysfunction and dopaminergic neuron vulnerability in PD.

## 4 Discussion

MSCs are sourced from bone marrow [40], adipose tissues [41], exfoliated deciduous tissues [42], and perinatal tissues like placenta and umbilical cord [43, 44, 45, 46]. Their ability to differentiate into dopaminergic neurons is essential for creating a PD cellular model. These MSCs have been used to generate dopaminergic neurons, showing promise for PD therapy [10, 47, 48]. Perinatal tissues, particularly placenta and umbilical cord, are advantageous due to easy procurement, minimal ethical issues, and underutilization in model development. Thus, we selected Wharton's Jelly (WJ) from the umbilical cord for this study due to its properties and feasibility. Previous research indicates WJMSCs can differentiate into dopaminergic neurons in both 2D and 3D cultures [49, 50, 51]. Therefore, we used WJMSCs to develop an in vitro PD‐like model. According to ISCT, isolated MSCs must meet three criteria: plastic adherence, expression of markers like CD90, CD73, and CD105, absence of markers such as CD34 and CD45, and the ability to differentiate into adipocytes, osteocytes, and chondrocytes [52]. WJMSCs isolated enzymatically displayed fibroblast‐like morphology, expressed positive MSC markers, lacked negative markers, and differentiated into all three lineages, fulfilling ISCT criteria.

Numerous studies have shown that MSCs can differentiate into dopaminergic neurons using various protocols [8, 53, 54]. However, a thorough characterization of these neurons remains incomplete. Reports indicate that WJMSCs have been induced into neuronal and dopaminergic lineages using a mix of compounds, small molecules, and growth factors like β‐mercaptoethanol (βME), basic fibroblast growth factor (bFGF), FGF8, sonic hedgehog (Shh), IBMX, retinoic acid, N2, butylated hydroxyanisole, and dimethyl sulfoxide [9, 10, 55]. The differentiation period varies from minutes to weeks, depending on MSCs and inducing agents [56]. Our study aimed to create a rapid protocol ensuring the stability of differentiated cells by using antioxidants, chromatin modifiers, and small molecules to induce WJMSCs into the dopaminergic lineage. This method resulted in cells with neuronal stem cell‐like morphology and expressing mature neuronal and dopaminergic markers within 24 h, persisting for up to 6 days. By the 8th day, dopaminergic expression diminished, although neuronal morphology remained. Dopaminergic neurons must express markers such as Nurr1, DAT, DDC, and TH. Nurr1, a nuclear receptor, is critical for developing and maintaining midbrain neurons [57, 58, 59]. TH is the rate‐limiting enzyme in dopamine synthesis, a neurotransmitter involved in motor control and emotional regulation, converting L‐tyrosine to L‐DOPA. DDC then converts L‐DOPA to dopamine [60, 61]. For functional characterization, we also examined transporters like DAT and KCNJ6. DAT regulates intracellular dopamine levels and neurotransmission, primarily found on SN neurons, marking dopaminergic neurons, while KCNJ6 stabilizes the membrane potential and modulates dopaminergic neuron effects [62, 63, 64, 65]. The robust expression of these dopaminergic markers confirmed that the differentiated WJMSCs were genuinely dopaminergic.

For neurodegeneration of DAN, we have utilized the MPP+ iodide and A53T variant of α‐SYN. MPTP crosses the blood–brain barrier and is oxidized by type B monoamine oxidase into its toxic form, MPP+, leading to dopaminergic neuron degeneration [34]. Notably, MPP+ exposure replicates PD pathology features, including alpha‐synuclein aggregation [66, 67]. Although rare, the A53T mutation's association with early‐onset PD highlights the importance of understanding its mechanism for models and therapies, especially since α‐SYN, a key player in this mutation, is a risk factor for sporadic PD, with its aggregates forming Lewy bodies in both sporadic and familial cases [11, 21]. Our model showed increased SNCA gene and α‐SYN protein expression upon MPP+ and A53T‐induced degeneration, confirming neurodegeneration. Nurr1 plays a crucial role in the development and differentiation of dopaminergic neurons in the SNpc by controlling the expression of key genes such as TH, DAT, and vesicular monoamine transporter (VMAT). This regulatory function is particularly relevant in PD, as recent studies have shown. Dopaminergic neurons in the SNpc of PD patients exhibit decreased Nurr1 expression [68, 69]. The significance of Nurr1‐regulated genes, like TH, in maintaining dopaminergic neuron health is further emphasized by PD pathology research. TH is essential for dopaminergic neuron survival and function, as its absence results in reduced dopamine production and PD [70]. As such, sustained TH presence is vital for dopaminergic neuron survival and functionality [71]. Although TH is vital for dopamine synthesis, other genes regulated by Nurr1, such as DAT, also contribute significantly to dopaminergic neuron function and PD pathogenesis. The modulation of DAT function by α‐SYN is implicated in PD pathogenesis, highlighting its role in dopaminergic neurodegeneration [72]. Upon degeneration of DAN, a significant decrease in these dopaminergic markers was observed, further validating neuronal degeneration by MPP+ and A53T exposure.

Although the molecular mechanisms involved in SNpc neuron degeneration are not fully understood oxidative stress and dysfunctional mitochondrial membrane potential are some of the factors [22, 23]. Interrupting the flow of electrons along the respiratory chain and interfering with various metabolic processes within the mitochondria can generate ROS and reactive nitrogen species (RNS), thereby exacerbating oxidative stress [73]. As mentioned previously, MPP+ exposure interrupts the supply of cell energy, which leads to an increase in ROS levels, indicating oxidative stress. This leads to mitochondrial dysfunction, further highlighting the role of oxidative stress in neuronal death [74, 75, 76, 77, 78, 79, 80]. This focused action on mitochondrial function provides a clearer framework for studying mitochondrial impairment in PD. In contrast, rotenone also inhibits complex I but affects other pathways, introducing experimental variability, while 6‐OHDA targets dopaminergic neurons with broader neurotoxic effects [81]. Reportedly, A53T showed increased ROS production in dysfunctional mitochondria compared to WT or A30P [82]. Furthermore, studies have reported higher levels of RNS in PD patients, evident from increased levels of nitrite/nitrate in blood and spinal fluid [83, 84, 85]. MPP+ exposure has been associated with alterations in NO levels and nitrites, and neuronally derived NO has been reported to partially mediate MPTP‐induced neurotoxicity, underscoring NO's role in dopaminergic neurodegeneration [86, 87]. Additionally, nitrated α‐SYN, a hallmark protein in PD pathology, has been reported to accelerate the degeneration of nigral dopaminergic neurons, further emphasizing the role of nitration processes in PD progression [88]. In agreement with these findings, we also observed significantly higher ROS generation and nitrite production upon induction with MPP+ and A53T, underscoring their role in neurodegeneration.

Mitochondrial dysfunction occurs early and acts causally in PD pathogenesis, based on evidence from genetic and toxin models of experimental PD [89, 90, 91]. Mutated and aggregated α‐SYN directly contributes to mitochondrial dysfunction in PD, implicating PD‐related gene products in α‐SYN‐mediated mitochondrial dysfunction [92]. A53T induces oxidative stress and affects mitochondria. It impairs its membrane potential as well as respiratory function, leading to mitochondrial autophagy and ultimately neurodegeneration [93]. Upon addition of A53T and MPP+, increased ROS and nitrite production can be correlated with reduced mitochondrial membrane potential, ultimately leading to neurodegeneration in DAN, as observed in our study. For protection of cells from oxidative damage, GSH is an important player. It is involved in the detoxification process and helps in maintaining the redox balance in the dopaminergic neurons by scavenging free radicals. Alteration in the homeostasis of GSH might lead to oxidative stress [94]. In the SNpc region of the brains of PD patients, decreased levels of GSH with increased oxidative stress have been observed [95, 96]. Likewise, in our model, a reduction of GSH was observed, which indicates that the neurons are susceptible to oxidative stress‐mediated damage and the antioxidant defense mechanism has been impaired in the DaN. Thus, our model effectively mimics PD‐like characteristics. While our model effectively represents key aspects of PD pathogenesis, it has limitations, such as limited cellular diversity, genetic variability, and simplified pathological complexity, as PD is a multifactorial disorder. To overcome these challenges, we will incorporate models addressing genetic mutations (e.g., LRRK2, PINK1, Parkin), utilize glial co‐cultures, and integrate other PD‐relevant stressors.

## 5 Conclusion

A cost‐effective, rapid, and effective model was developed to understand PD in this study. Our protocol for differentiating WJMSCs to DAN was stable and was able to generate functional DAN in a lesser duration. The PD model was successfully created by inducing oxidative stress via MPP+ and α‐SYN‐A53T and exhibited the molecular characteristics of PD. This in vitro model does not require any genetic modifications, and there are not complexities involved in the case of iPSCs or ESCs. Pharmaceutical companies, as well as various countries, are now stressing more on the use of in vitro models and less dependency on animals for drug discovery, screening, and development. This PD model can be used as a drug screening platform as well for screening and evaluating the efficacy of emerging therapeutic PD drugs at a large scale. Additionally, this model can also be employed to study signaling pathways involved in PD pathogenesis, to uncover their roles in neurodegeneration and neuronal survival.

## Author Contributions

Conceptualization: Naisarg Gamit and Sudha Warrier. Methodology: Naisarg Gamit. Software: Naisarg Gamit. Validation: Naisarg Gamit and Sudha Warrier. Formal analysis: Naisarg Gamit, Manasi Patil, B.S. Soumya, and Sudha Warrier. Investigation: Sudha Warrier. Resources: Sudha Warrier; Data curation: Naisarg Gamit and Sudha Warrier. Writing – original draft preparation: Naisarg Gamit and Sudha Warrier. Writing – review and editing: Naisarg Gamit, Manasi Patil, B.S. Soumya, Arun Dharmarajan, and Sudha Warrier. Visualization: Naisarg Gamit and Sudha Warrier. Supervision: Sudha Warrier. Project administration: Sudha Warrier. Funding acquisition: Sudha Warrier.

## Conflicts of Interest

The authors declare no conflicts of interest.

## Supporting information


Figure S1.

Table S1.

## Data Availability

All the data presented in this study are included in this article. All the other relevant data may also be available from the corresponding author upon a reasonable request.
