# Supplementary figures and images for: Development of In Vitro Parkinson's Disease Model Mediated by MPP+ and α‐Synuclein Using Wharton's Jelly Mesenchymal Stem Cells
Source: CNS Neurosci Ther. 2025 Apr 22;31(4):e70299. doi: 10.1111/cns.70299 (PMC12012574; doi:10.1111/cns.70299)

Figure S1. Full unedited gel for Figure 1b.

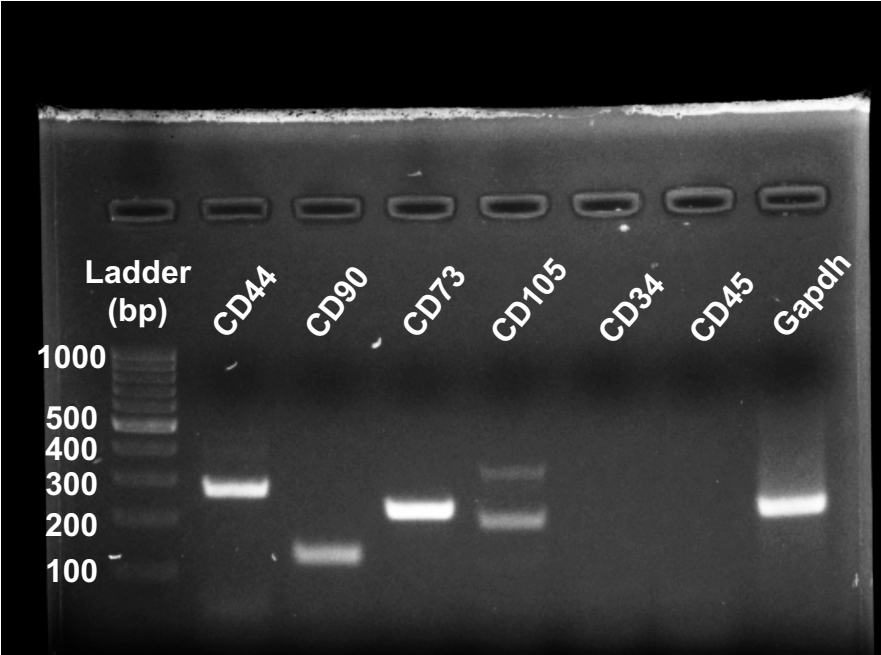

Supplement: Supplementary file 1 — Figure S1. [file CNS-31-e70299-s001.pdf]
