# Supplementary material for: Development of In Vitro Parkinson's Disease Model Mediated by MPP+ and α‐Synuclein Using Wharton's Jelly Mesenchymal Stem Cells
Source: CNS Neurosci Ther. 2025 Apr 22;31(4):e70299. doi: 10.1111/cns.70299 (PMC12012574; doi:10.1111/cns.70299)
Supplement: Supplementary file 2 — Table S1. [file CNS-31-e70299-s002.pdf]

Table S1. List of antibodies

Sr. No.	Antibody name	RRID/Cat no.	Manufacturer	Dilution
1	CD44	RRID: AB_465045	Thermo Fisher Scientific, CA, USA	1:500
2	CD105	RRID: AB_467417	Thermo Fisher Scientific, CA, USA	1:500
3	Vimentin	Cat# CSB-PA004450	Cusabio Technologies LLC, TX, USA	1:500
4	CD90	RRID: AB_395970	BD Biosciences, CA, USA	1:500
5	CD34	RRID: AB_1210472	Thermo Fisher Scientific, CA, USA	1:500
6	CD45	RRID: AB_11063696	Thermo Fisher Scientific, CA, USA	1:500
7	Synapsin I	RRID: AB_2536207	Thermo Fisher Scientific, CA, USA	1:500
8	Tuj1	RRID: AB_2532242	Thermo Fisher Scientific, CA, USA	1:500
9	MAPT	Cat# PAB983Hu01	Cloud-Clone Corp., TX, USA	1:500
10	Nurr1	Cat# PAD964Hu01	Cloud-Clone Corp., TX, USA	1:500
11	TH	RRID: AB_2539844	Thermo Fisher Scientific, CA, USA	1:500 (IF); 1:1000 (ELISA)
12	alpha synuclein	RRID: AB_2532379	Thermo Fisher Scientific, CA, USA	1:500 (IF); 1:1000 (ELISA)
13	CD73	RRID: AB_10596508	Thermo Fisher Scientific, CA, USA	1:500
14	CD44	Cat# ab9524	Abcam, USA	1:500
15	anti-rabbit Alexa Fluor 488	RRID: AB_2633280	Thermo Fisher Scientific, CA, USA	1:1000
16	anti-rabbit Alexa Flour 594	RRID: AB_2534095	Thermo Fisher Scientific, CA, USA	1:1000
17	anti-mouse Alexa Fluor 488	RRID: AB_2534088	Thermo Fisher Scientific, CA, USA	1:1000
18	anti-mouse Alexa Flour 594	RRID: AB_2534091	Thermo Fisher Scientific, CA, USA	1:1000
19	anti-rabbit IgG HRP	RRID: AB_1185567	Thermo Fisher Scientific, CA, USA	1:1000
